# The effects of elemene emulsion injection on rat fecal microbiota and metabolites: Evidence from metagenomic exploration and liquid chromatography-mass spectrometry

**DOI:** 10.3389/fmicb.2022.913461

**Published:** 2022-11-24

**Authors:** Lei Gu, Hao Wu, Yang Zhang, Yousheng Wu, Yuan Jin, Tian Li, Litian Ma, Jin Zheng

**Affiliations:** ^1^Department of Cardiology, Xi'an International Medical Center Hospital Affiliated to Northwest University, Xi'an, China; ^2^Department of Traditional Chinese Medicine, Tangdu Hospital, Air Force Medical University, Xi'an, China; ^3^Health Center of 95816 of the People's Liberation Army, Wuhan, China; ^4^National Demonstration Center for Experimental Preclinical Medicine Education, Air Force Medical University, Xi'an, China; ^5^Department of Internal Medicine, The Third Affiliated Hospital of Xinxiang Medical College, Xinxiang, China; ^6^School of Basic Medicine, Fourth Military Medical University, Xi'an, China; ^7^Department of Gastroenterology, Tangdu Hospital, Air Force Medical University, Xi'an, China

**Keywords:** elemene emulsion injection, fecal microbiota, metabolomics, 16S rRNA, metagenome

## Abstract

**Objective:**

Elemene emulsion injection (EEI) has been approved for interventional and intracavitary chemotherapy in treating malignant ascites in China, but few studies have focused on the effects of EEI on gut microbiota and metabolites. In this study, we investigated the effects of EEI on the fecal microbiota and metabolites in healthy Sprague-Dawley (SD) rats.

**Methods:**

We randomly assigned 18 male SD rats to three groups (*n* = 6 in each group): the sham group (group S), the low-concentration EEI group (L-EEI), and the high-concentration EEI group (H-EEI). The L-EEI and H-EEI rats were administered 14 days of consecutive EEI, 20 mg/kg, and 40 mg/kg intraperitoneally (IP). Group S rats were administered the same volume of normal saline. On day 14, each animal's feces were collected for metagenomic sequencing and metabolomic analysis, and the colonic contents were collected for 16S rRNA sequencing.

**Results:**

EEI could alter the β-diversity but not the α-diversity of the fecal microbiota and induce structural changes in the fecal microbiota. Different concentrations of EEI affect the fecal microbiota differently. The effects of different EEI concentrations on the top 20 bacteria with significant differences at the species level among the three groups were roughly divided into three categories: (1) A positive or negative correlation with the different EEI concentrations. The abundance of *Ileibacterium Valens* increased as the EEI concentration increased, while the abundance of *Firmicutes bacteria* and *Clostridium sp. CAC: 273* decreased. (2) The microbiota showed a tendency to increase first, then decrease or decrease first, and then increase as EEI concentration increased—the abundance of *Prevotella sp. PCHR, Escherichia coli*, and *Candidatus Amulumruptor caecigallinarius* tended to decrease with L-EEI but significantly increased with H-EEI. In contrast, L-EEI significantly increased *Ruminococcus bromii* and *Dorea sp. 5–2* abundance, and *Oscillibacter sp. 1–3* abundance tended to increase, while H-EEI significantly decreased them. (3) L-EEI and H-EEI decreased the abundance of bacteria (*Ruminococcaceae bacterium, Romboutsia ilealis*, and *Staphylococcus xylosus*). Fecal metabolites, like microbiota, were sensitive to different EEI concentrations and correlated with fecal microbiota and potential biomarkers.

**Conclusion:**

This study shows that intraperitoneal EEI modulates the composition of rat fecal microbiota and metabolites, particularly the gut microbiota's sensitivity to different concentrations of EEI. The impact of changes in the microbiota on human health remains unknown, particularly EEI's efficacy in treating tumors.

## Introduction

Elemenes are sesquiterpene compounds derived from the traditional Chinese medicinal plant, *Curcuma wenyujin*. Elemene emulsion injection (EEI) contains the active ingredients β-, γ-, and δ-elemene and excipients such as soybean lecithin, cholesterol, ethanol, disodium hydrogen phosphate, and sodium dihydrogen phosphate. The National Medical Products Administration of China has approved it for interventional and intracavitary chemotherapy and for treating cancerous pleural ascites. Furthermore, combining EEI with conventional radiotherapy or chemotherapy could improve their therapeutic effects (Jiang et al., 2017; Tong et al., 2020) against lung cancer (Chen et al., 2020), nasopharyngeal cancer, brain tumors (Liu S. et al., 2020), bone metastases, and others (Cai et al., 2021) while decreasing their adverse effects (Chen et al., 2020; Liu S. et al., 2020; Hashem et al., 2021).

Elemene-containing hyperthermic intraperitoneal chemotherapy has been used to treat peritoneal metastatic advanced gastric cancer with minimal myelosuppression (Zheng et al., 2014). Recent studies have shown that gut microbiota is critical to human health (Markowiak and Slizewska, 2017; Singh et al., 2017; Sommer et al., 2017; Gentile and Weir, 2018). Natural selection and adaptation maintain the microbiota-host-environment system in dynamic equilibrium, creating mutual constraints (Smits et al., 2013). Studies have linked gut microbiota to gastric and breast cancers (Chen et al., 2019). In addition, long-term antibiotic use can change gut microbes, increasing the risk of colorectal and prostate cancers (Boursi et al., 2015a,b; Dik et al., 2016; Ianiro et al., 2016; Chen et al., 2019). Elemene is an anticancer drug with unknown effects on the gut microbiota. The only study showing that β-elemene improves brain metabolites in obese C57BL/6 male mice fed a high-fat diet (HFD) and reversed HFD-induced changes in gut bacterial composition and content in mice (Zhou et al., 2021). Based on the above evidence, it is not yet possible to link the neuroprotective effect of EEI to the intestinal microbiota, nor can it be proved that there is a causal relationship between gut microbiota and EEI on neuroprotective effects. Likewise, it is not yet known whether the regulation of the gut microbiota would affect the therapeutic effect of EEI on tumors and the protective effect on nerves. Therefore, this study used meta-genomic sequencing and untargeted metabolomics techniques to explore the effects of different EEI concentrations on the rat fecal microbiota and metabolites and provide evidence that EEI administration could regulate the microbiota and metabolites in the rat.

## Materials and methods

### Animals

Eighteen male Sprague-Dawley (SD) rats (weighing 220–250 g, aged 9–11 weeks) were acquired from the Air Force Medical University under certificate number SCXK (Shaan) 2019-001. The rats were kept in separate cages, each with three rats. A 12-h light/dark cycle was implemented in the rearing environment, with a temperature of 20–25 °C and a relative humidity of 50–65%. The Animal Welfare and Ethics Committee, Laboratory Animal Center, and Air Force Military Medical University (IACUC-20220522) approved this study. All animal procedures were performed following the US National Institutes of Health's (NIH) Guide for the Care and Use of Laboratory Animals (NIH publication no. 85–23, revised).

### Materials

EEI was bought from Huali Jingang Pharmaceutical Co., Ltd. (Liaoning, China). According to literature reports, the detection data of 24 batches of samples showed that the content of β-syringene was 6.0 to 8.4%, with an average value of 7.2%, the content of β-elemene chiral isomer (RRT about 0.96) was 3.2–4.6%, with an average value of 3.9%, and the total of the two was 9.2–12.7%, with an average of 11.1% (Zhihua, 2018). The NEXTFLEX Rapid DNA-Seq Kit (Bioo Scientific, USA) and NovaSeq Reagent Kits/HiSeq X Reagent Kits (Illumina, America) were used as the primary kits for metagenomic sequencing. The main reagents and instruments for liquid chromatography-mass spectrometry (LC-MS) untargeted metabolomics include an ultra-high pressure liquid chromatography (UHPLC) liquid chromatography system (Vanquish Horizon system, Thermo), a mass spectrometer (Q-Exactive, Thermo), methanol and acetonitrile (Fisher Chemical), 2-propanol (Merck), and 2-Chloro-L-Phenylalanine (Adamas-beta).

### Grouping and administration

Eighteen male SD rats were randomly divided into three groups of six rats each: the sham group (group S), the low-concentration EEI group (L-EEI), and the high-concentration EEI group (H-EEI). Before the experiment started, rats were fed adaptively for 1 week and had free access to water and food. Different EEI concentrations were selected based on previously published studies (Ma et al., 2021; Sun et al., 2022; Wu et al., 2022). For 14 days, L-EEI rats received elemene emulsion [20 mg/(kg·d)] intraperitoneally (IP), H-EEI rats received elemene emulsion [40 mg/(kg·d)] IP, and group S rats received the same volume of normal saline IP as H-EEI. To equalize volume, L-EEI rats were supplemented with normal saline to equal the volume in H-EEI before injection.

### Amplification of the 16S rRNA gene and sequencing of colon contents

Fresh colon contents samples from 3 groups (*n* = 18) were collected in cryogenic vials and stored immediately in liquid nitrogen. Amplification and sequencing of 16S rRNA genes of colon contents were performed on the Illumina platform (Illumina, San Diego, USA) according to the standard protocols of Majorbio Bio-Pharm Technology Co. Ltd. (Shanghai, China). The V3–V4 region of the bacterial 16S rRNA gene was amplified with primer pairs 338F (5'-ACTCCTACGGGAGGCAGCAG-3') and 806R (5'-GGACTACHVGGGTWTCTAAT-3') (Liu et al., 2016). Raw FASTQ files were de-multiplexed using an in-house Perl script, then quality-filtered by Fast version 0.19.6 (Chen et al., 2018) and merged by FLASH version 1.2.7 (https://ccb.jhu.edu/software/FLASH/index.shtml) (Mago and Salzberg, 2011) with the following criteria: (1) The 300 bp reads were truncated at any site receiving an average quality score of <20 over a 50 bp sliding window, and the truncated reads shorter than 50 bp were discarded; reads containing ambiguous characters were also discarded. (2) Only overlapping sequences longer than 10 bp were assembled according to their overlapped sequence. The maximum mismatch ratio of the overlap region is 0.2. Readings that could not be assembled were discarded. After that, operational taxonomic units (OTUs) clustering analysis and taxonomic analysis were performed (UPARSE, version 7.0.1090, http://drive5.com/uparse/) (Edgar, 2013). OTU clustering was performed on non-repetitive sequences (excluding single sequences) according to 97% similarity, and the chimeras were removed in the clustering process to obtain the representative sequences of OTUs. The sequences with more than 97% similarity to the representative sequences were selected, and the OTU table was generated. To obtain the corresponding species classification information of each OTU, compare the following databases: the bacterial and archaeal 16S rRNA databases [Silva (Release 138 http://www.arb-silva.de) and Greengene (Release 13.5 http://greengenes.secondgenome.com/)]. First, the α-diversity analysis (http://www.mothur.org/wiki/Calculators) of colonic contents was assessed by community richness (ACE, Sobs, and Chao), community diversity (Shannon and Simpson), and community coverage (coverage) (Mothur, version 1.30.2, https://mothur.org/wiki/calculators/). Based on the results of OTU clustering analysis, a Venn diagram was used to display the common and unique microbiota among the three groups (R, version 3.3.1) at the genus level. Second, the composition and relative abundance were analyzed at the genus level in each group (R, version 3.3.1). Partial least squares discriminant analysis (PLS-DA) (Gromski et al., 2015) was used to analyze the similarity among the three groups at the genus level (http://fiehnlab.ucdavis.edu/staff/kind/Statistics/Concepts/OPLS-PLSDA, R, version 3.3.1). Permutational multivariate analysis of variance (PERMANOVA) (Kelly et al., 2015) was used to analyze the degree of explanation of different grouping factors on differences between samples (Bray-Curtis). Third, the non-metric multidimensional scaling (NMDS) was used to evaluate the β-diversity of colon contents among three groups at the genus level.

### Metagenomic sequencing and assembly

Total genome DNA was extracted from 200–300 mg of stool samples. Before analyzing the raw data generated using the Illumina sequencing platform (Illumina, San Diego, USA), the software Fast (https://github.com/OpenGene/fastp) was used to perform statistical and quality control on the raw data to ensure subsequent analysis accuracy. About 300 bp of fragments were sequenced in metagenomics. Supplementary Table 1 lists the original sequence and clean data statistics. Then, the software BWA v0.7.17 was used to decontaminate the sample's host genome. The host name was Vertebrates: *rattus_norvegicus*. Supplementary Table 1 lists the data after removing the host genome. Sequences of different sequencing depths were assembled using tEGAHIT v1.1.2 (https://github.com/voutcn/megahit) (Li et al., 2015). After splicing and assembly, the shortest contig length retained was 300 bp. Supplementary Table 2 lists the data after assembly. Afterward, an open reading frame (ORF) prediction was performed on the contigs in the splicing results using Prodigal (https://github.com/hyattpd/Prodigal). Genes with a nucleic acid length ≧ of 100 bp were selected and translated to amino acid sequences to generate a statistical table of gene prediction results for each sample (Supplementary Table 3). CD-HIT software (http://www.bioinformatics.org/cd-hit/) (Fu et al., 2012) was used to cluster the predicted gene sequences of all samples (the default parameters are 90% identity and 90% coverage). The representative sequence for constructing a gene set with no duplicate genes was the longest gene in each class. The SOAPaligner software (http://soap.genomics.org.cn/) (Li et al., 2009) was used to align each sample's high-quality reads with the non-redundant gene set (default parameter: 95% identity) and count the genes in the corresponding sample's abundance information. Gene abundance was calculated using reads per kilobase million (Lawson et al., 2017). The linear discriminant analysis effect size (LEfSe) differential discriminant analysis (http://huttenhower.sph.harvard.edu/galaxy/root?tool_id=lefse_upload) (Segata et al., 2011; Zhang et al., 2013) was used to identify the species that best explains the differences between groups in multiple samples.

### Taxonomic annotation and difference analysis

The non-redundant gene set was aligned with the non-redundant protein sequence database (NR database) using DIAMOND (Buchfink et al., 2015, 2021) (https://github.com/bbuchfink/diamond) (parameters: BLASTP; E-value ≤ 1 × 10^−5^). The taxonomic information database correlating to the NR database was used to get species annotation results. The species abundance was calculated by adding up the abundance of its genes. Venn plots were used to count shared and unique species across multiple groups, and community column charts were used to visually study the dominant species in a community.

### Fecal metabolomics

Different groups of fecal metabolites were detected using LC-MS, and metabolites with differential expressions were found. First, a 50 mg sample was weighed accurately, and then 400 μL of extraction solution [methanol: water = 4:1 (v: v)] containing 0.02 mg/mL of the internal standard (L-2-chlorophenyl alanine) was added. Second, the sample was ground with a frozen tissue grinder for 6 min (−10°C, 50 Hz), and ultrasonic extraction was used for 30 min (5°C, 40 kHz) at low temperature. Third, let the sample stand at −20°C for 30 min, centrifuge for 15 min (13,000 *g*, 4°C), and then transfer the supernatant to the inlet with an inner cannula. In addition, 20 μL of supernatant was pipetted from each sample and mixed as a quality control sample (Zheng et al., 2021; Zhu et al., 2022).

The raw data were imported into the metabolomics processing software ProgenesisQI (Waters Corporation, Milford, USA) for baseline filtering, peak identification, integration, retention time correction, peak alignment, and other tasks before a data matrix containing retention time, mass-to-charge ratio, and peak intensity was obtained. The software was then used to perform a library search of characteristic peaks, matching the MS and MS/MS mass spectral information to the metabolic database. The software was then used to search for characteristic peaks in a library by matching the information from the MS and MS/MS mass spectra to the metabolic database. The MS mass error was set to less than 10 ppm, and metabolites were identified based on secondary mass spectrometry matching scores. The main databases are http://www.hmdb.ca/ and https://metlin.scripps.edu/. The total ion chromatograms of the quality control samples in positive and negative ion modes show that the peak shape is acceptable, and the distribution is relatively uniform under this detection condition (Supplementary Figure 3). The PLS-DA model quality parameters and the corresponding permutation test (R2 intercept, 0.8907; Q2 intercept, −0.7653) demonstrate the statistical validity of the analysis and indicate distinct metabolic profiles among the three groups (Supplementary Figure 2).

Principal component analysis (Worley and Powers, 2013) was used to identify the “main” elements and structures in the data to reduce the dimensionality of the high-dimensional data space while minimizing the loss of data information. We also used the PLS-DA (Gromski et al., 2015) method to reduce the dimensionality of the data to better obtain the different information between groups. To prevent overfitting in the PLS-DA, we used the permutation test to determine whether the PLS-DA model is overfitting. The Kruskal-Wallis (H) test was used to compare differential metabolites among multiple groups. The metabolites that were common or unique among the differential metabolites between the two groups were displayed using Venn diagrams.

### Association of fecal differential metabolites with fecal microbiota

The *Pearson* correlation analysis found correlation coefficients between fecal microbiota and metabolites. The correlation between significantly differential fecal microbiota within the genus (*P* < 0.01) and differential metabolites (*P* < 0.01) is shown in **Figure 6**. The clustering algorithm was hierarchical clustering, the distance algorithm was Euclidean, and the hierarchical clustering method was complete (took the distance between the two data points that were the farthest from the two combined data points as the distance between the two combined data points).

### Association of fecal differential metabolites and metabolic pathways

Using Pearson correlation analysis, we perform a metabolic pathway study of our metagenomic data and link it to the chemicals identified in the stool samples. The correlation between significantly differential metabolites (*P* < 0.01) and significantly differential metabolic pathways (*P* < 0.01) in level 3 of the Kyoto Encyclopedia of Genes and Genomes (KEGG, https://www.kegg.jp/) (**Figure 7**).

### Statistical analysis

Sequencing was performed on the rats' feces (*n* = 18). The Wilcoxon rank-sum test was used to calculate α-diversity among three groups, and the results were expressed as mean ± standard deviation. *P* < 0.05 was deemed significant. The Kruskal-Wallis test (H test) was used to compare the differential microbiota among multiple groups, and the Tukey-Kramer procedure was used for *post-hoc* testing. *P* < 0.05 was deemed significant. The Kruskal-Wallis test and Scheffe's *post*-hoc test were also used to find differences in metabolites between more than two groups. *P* < 0.05 was considered significant (Edwards and Berry, 1987; Lee and Lee, 2018).

## Results

### H-EEI altered the β-diversity of fecal microbiota

**Figure 1:**
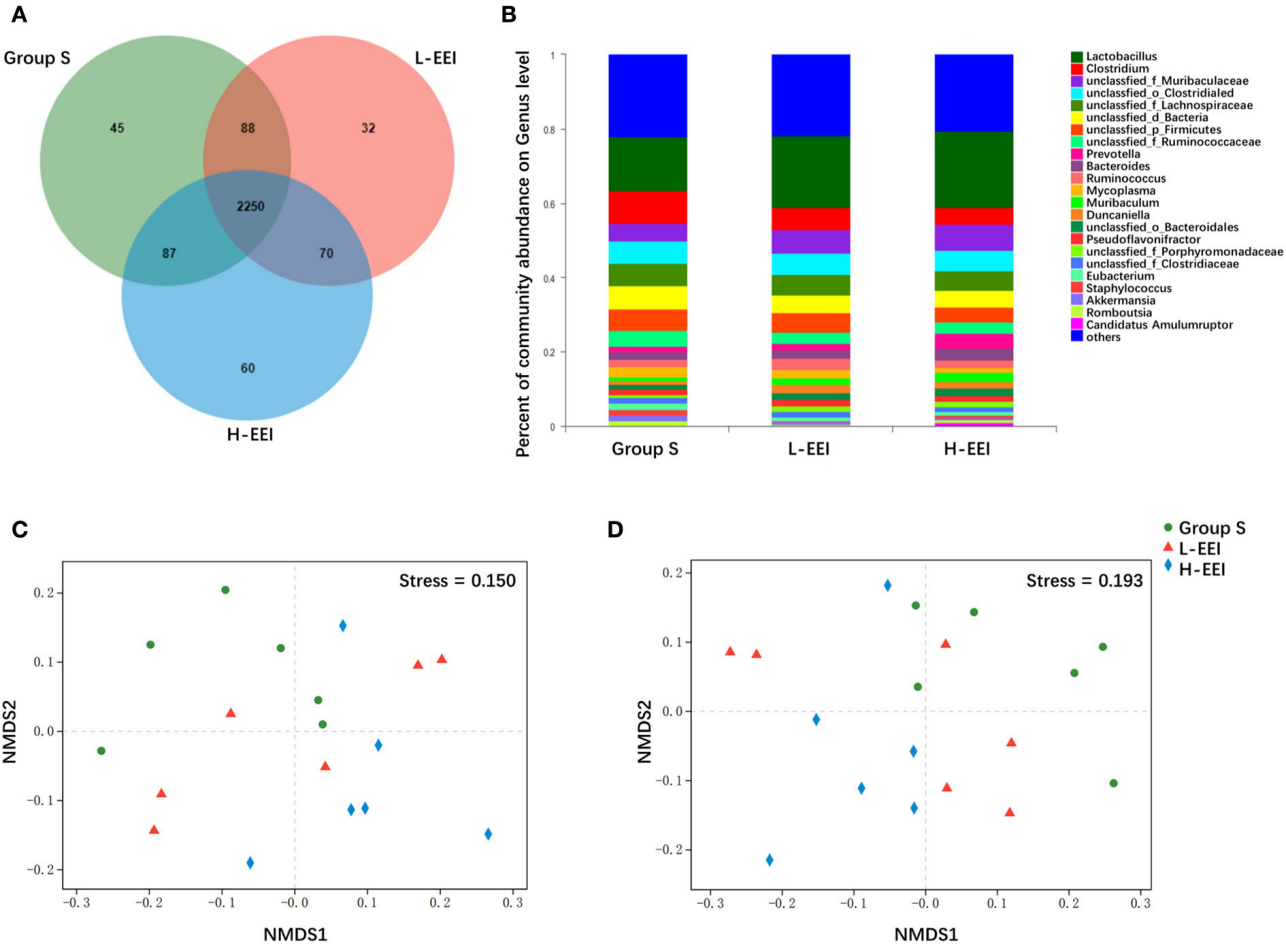
H-EEI altered the β-diversity of fecal microbiota. **(A)** The Venn diagrams show that bacteria that are unique and shared by different groups at the genus level. The overlapping part represents the bacteria shared by different sample groups, the non-overlapping part represents the bacteria unique to the sample group, and the number represents the corresponding species. There were 45 and 32 genera specific to groups S and L-EEI, respectively, and 60 genera specific to H-EEI. **(B)** The relative abundance of the genus level in each group is shown. The horizontal axis represents the sample name, and the vertical axis represents the proportion of microbiota in the sample. Colored columns represent different microbiota, and column length represents a proportion of the microbiota. **(C,D)** The NMDS analysis of different groups shows a distinct formation of clusters at the genus level **(C)**, and according to species level **(D)**, Bray–Curtis. NMDS is a data analysis method that simplifies research objects in multidimensional space into low-dimensional space for positioning, analysis, and classification while retaining the original relationship between objects. The pros and cons of NMDS analysis results are measured by stress. It is generally believed that when stress <0.2, it can be represented by a two-dimensional point graph of NMDS, and this graph has a certain explanatory significance. The distance between the points indicates the degree of difference, and the horizontal and vertical coordinates indicate the relative distance, which has no practical significance. The data used in the analysis were derived from metagenomics sequencing data of fecal mirobiota (*n* = 6 in each group).

Different concentrations of elemene did not significantly affect the α-diversity of fecal microbiota (Supplementary Table 4). The Venn diagrams showed bacteria that are unique and shared among different groups at the genus level (Figure 1A). There were 45 and 32 genera specific to groups S and L-EEI, respectively, and 60 genera specific to H-EEI. Deeper taxonomic levels, including species, genera, and families, were investigated to learn more about the fecal microbiota of different groups. The relative abundance of the genus level in each group is shown in Figure 1B. The relative abundance of the species and families in each group is shown in Supplementary Figure 1. The NMDS analysis of different groups showed a distinct formation of clusters at the genus level (Figure 1C) and species level (Figure 1D). The differences between L-EEI and group S were less evident than those between H-EEI and group S, which formed quite different clusters. There is a clear visual distinction between group S and the H-EEI (stress = 0.150 for genera and stress = 0.193 for species).

### Distinctive sensitivity of fecal microbiota to different concentrations of EEI

**Figure 2:**
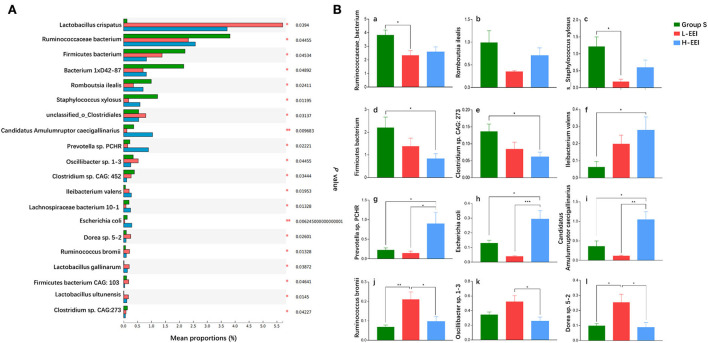
Distinctive sensitivity of fecal microbiota to different concentrations of EEI. **(A)** The abundance of the top 20 fecal bacteria with significant differences at the species level was determined using fecal metagenomics data (Kruskal–Wallis test, *P* < 0.05 was considered significant). The vertical axis represents the species names, the horizontal axis represents the percentage value of a species' abundance in the sample, and different colors represent different groups (green to group S, red to L-EEI, and blue to H-EEI). **(B)** *Post-hoc* analysis of the top 20 fecal bacteria with significant differences in **(A)** (Tukey–Kramer, *P* < 0.05 was considered significant). *n* = 6 in each group, *P < 0.05, **P < 0.01, ***P < 0.001.

We further compared the differences among the three groups to determine the effect of different concentrations of EEI on rat fecal microbiota. Figure 2A shows the abundance of the top 20 bacteria with significant differences at the species level among the three groups (Kruskal–Wallis test; *P* < 0.05 was considered significant). Following the multiple-group test, we compared the groups using pairwise comparisons (Tukey-Kramer, *P* < 0.05 was considered significant, Figure 2B). The effects of different EEI concentrations on the top 20 bacteria with significant differences at the species level among the three groups could be roughly divided into three categories: (1) A positive or negative correlation with the concentration of the EEI. The abundance of *Ileibacterium Valens* increased as the EEI concentration increased, while the abundance of *Firmicutes bacterium* and *Clostridium sp. CAC: 273* decreased. (2) The microbiota showed a tendency to increase first, then decrease or decrease first, and then increase as EEI concentration increased. Compared to group S, the abundance of *Prevotella sp. PCHR, Escherichia coli*, and *Candidatus Amulumruptor caecigallinarius* tended to decrease by L-EEI but significantly increased by H-EEI (*P* < 0.05). Inversely, L-EEI significantly increased the abundance of *Ruminococcus bromii* (*P* < 0.01) and *Dorea sp. 5–2* (*P* < 0.05), and then, *Oscillibacter sp. 1–3* tended to increase, but H-EEI significantly decreased the abundance of these three microorganisms. (3) Both L-EEI and H-EEI tend to decrease the abundance of the bacteria (*Ruminococcaceae bacterium, Romboutsia ilealis*, and *Staphylococcus xylosus*).

First, the abundance of *Firmicutes bacterium* and *Clostridium sp. CAC: 273* decreased gradually as EEI concentration increased, and the H-EEI was significantly lower than group S (*P* < 0.05). *Ileibacterium Valens* abundance increased gradually with the increasing EEI concentration, and the H-EEI was significantly higher than group S (*P* < 0.05). Second, for *Prevotella sp. PCHR, Escherichia coli*, and *Candidatus Amulumruptor caecigallinarius*, the L-EEI tended to decrease the abundance compared to group S, whereas the H-EEI significantly increased the abundance compared to the L-EEI (*P* < 0.05). Third, for *Ruminococcus bromii, Oscillibacter sp. 1–3*, and *Dorea sp. 5–2*, L-EEI tended to increase the abundance compared to group S. In contrast, H-EEI significantly decreased the abundance compared to L-EEI (*P* < 0.05). Finally, both L-EEI and H-EEI decreased the abundance of the *Ruminococcaceae bacterium, Romboutsia ilealis*, and *Staphylococcus xylosus*.

**Figure 3:**
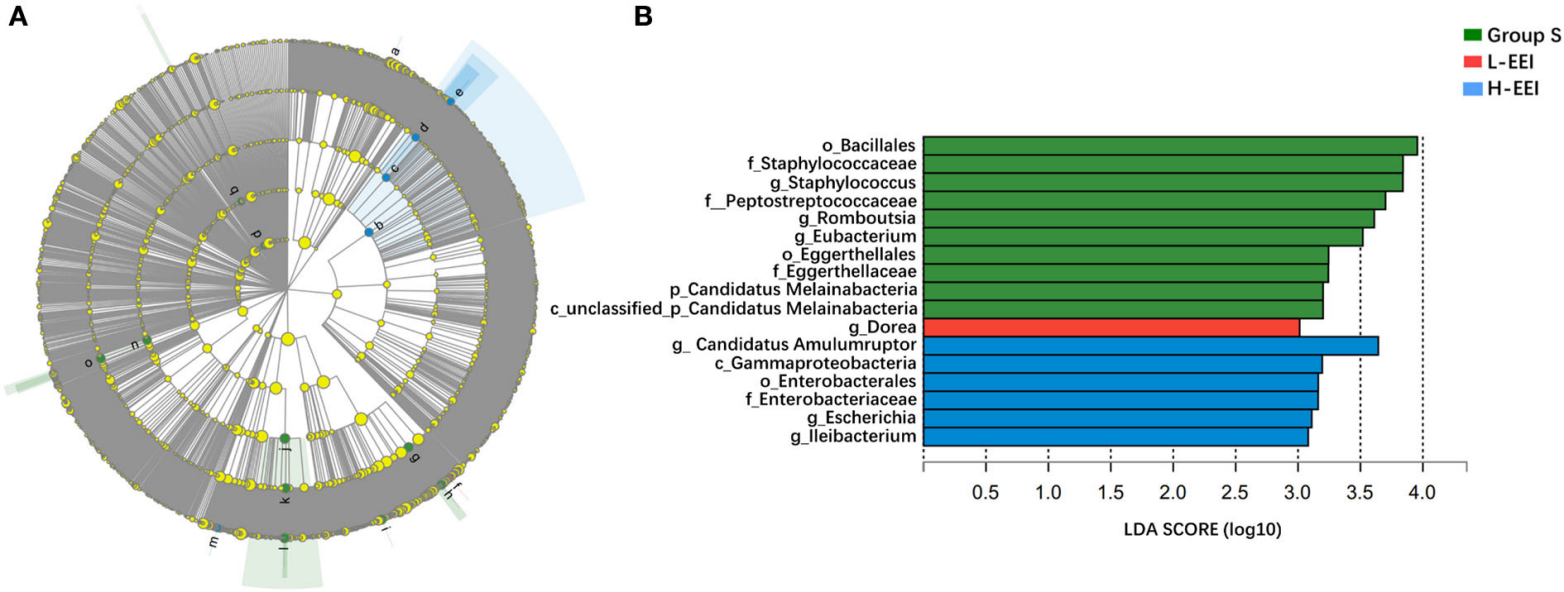
Biomarkers of L-EEI and H-EEI: g*_Dorea* and g*_Candidatus Amulumruptor*. **(A)** The hierarchy of linear discriminant analysis effect size (LEfSe) was based on fecal metagenomics data. Nodes in the figure with different colors represent microbial groups that are significantly enriched in the corresponding group and have a significant effect on the differences among the three groups; light yellow nodes represent microbial groups with no significant difference between groups or have no significant effect on the differences among the three groups. **(B)** The linear discriminant analysis (LDA) discriminant histogram. The higher the LDA score, the greater the influence of microbiota abundance on the differential effect. *P* < 0.05, LDA > 3.0.

LEfSe analysis was applied to investigate the biomarkers among the three groups. We found 17 differentially abundant taxa among the three groups, all with an LDA score > 3.0 and *P* < 0.05 (Figures 3A,B). LEfSe analysis identified ten genera with increased abundance in Group S, one genus with increased abundance in the L-EEI, and six genera with increased abundance in the H-EEI.

### L-EEI and H-EEI-induced differential fecal metabolites

**Figure 4:**
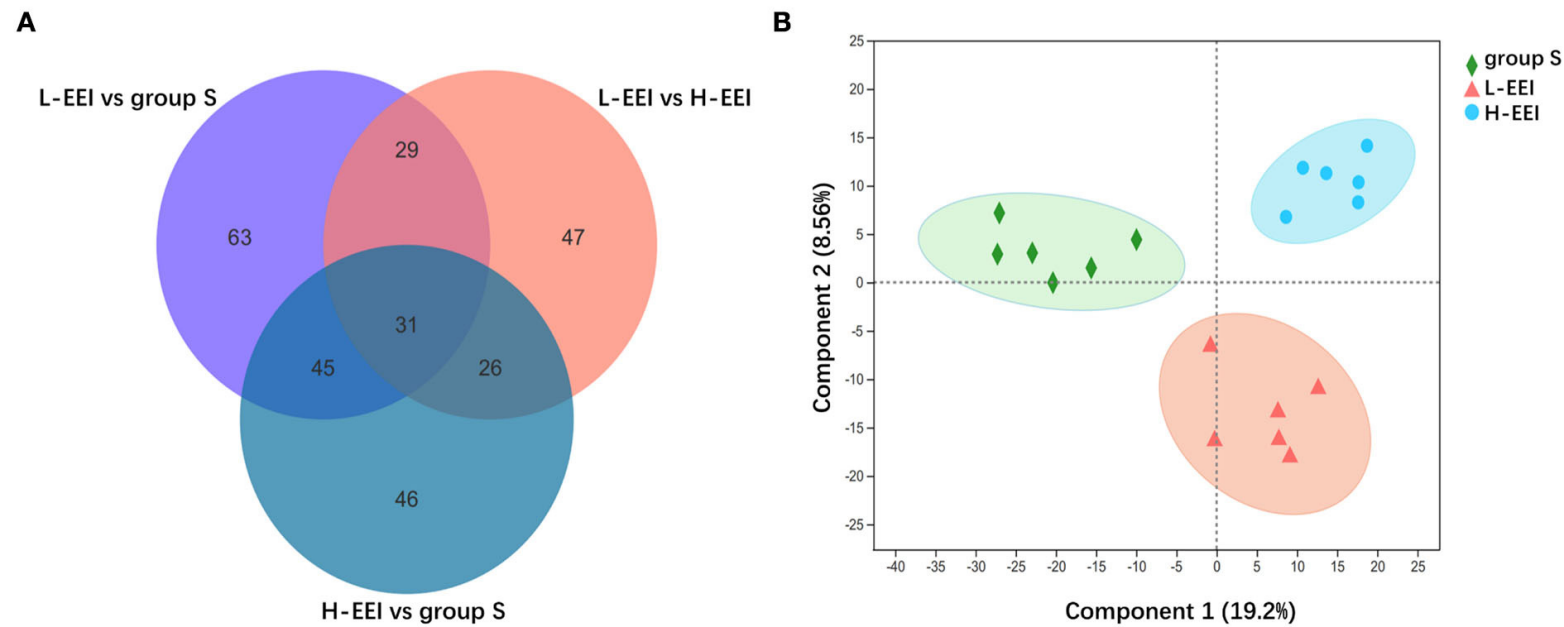
L-EEI and H-EEI induced differential fecal metabolites. **(A)** The common or unique metabolites are found after a pairwise comparison of three groups. In the figure, the overlapping part represents the number of metabolites shared by multiple metabolic sets, the non-overlapping part represents the number of metabolites unique to the metabolic set, and the number represents the number of corresponding metabolites. **(B)** PLS-DA score plot. The PLS-DA score map is used to visually represent the classification effect of the model. The greater the degree of separation among the three groups of samples in the figure, the more significant the classification effect. Component 1 is the first principal component explainability, and Component 2 is the second principal component explainability. The data used in the analysis were derived from LC-MS data of feces (*n* = 6 in each group).

We used LC-MS to directly test fecal metabolites to find out how different concentrations of EEI affected the metabolites of the fecal microbiota. Fecal metabolomics is the consequence of both host and microbiota interaction. The chemical signatures were identified based on internal standards (Majorbio Bio-Pharm Technology Co. Ltd.), the Kyoto Encyclopedia of Genes and Genomes (KEGG, http://www.genome.jp/kegg/), the Human Metabolome Database (HMDB 5.0, www.hmdb.ca) and METLIN 2019 (http://metlin.scripps.edu). The analysis of fecal metabolites revealed variations in different EEI concentrations. A total of 1,091 metabolites were identified, including vitamins and cofactors, peptides, nucleic acids, hormones and neurotransmitters, steroids, organic acids, lipids, and carbohydrates. A Venn diagram shows the common or unique metabolites found following a pairwise comparison of the three groups (Figure 4A). Figure 4B shows the findings of the PLS-DA model obtained from multivariate statistical comparisons of groups S, L-EEI, and H-EEI.

**Figure 5:**
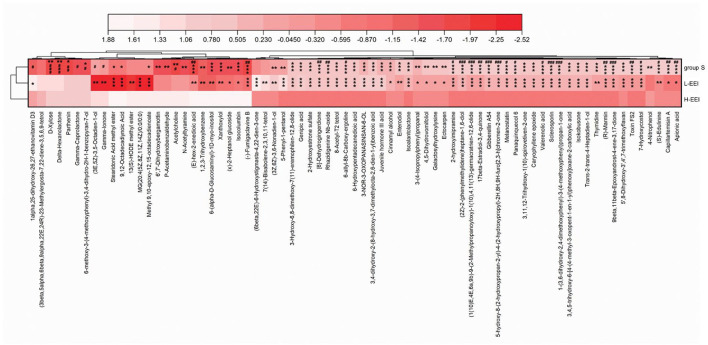
Differential metabolites among the three groups based on LC-MS data of feces. Darker colors indicate lower metabolite abundance, while lighter colors indicate higher metabolite abundance. The Kruskal–Wallis H test compares multiple groups, followed by the Scheffe *post-hoc* tests for metabolites with *P* < 0.01. *n* = 6 in each group, *P < 0.05, **P < 0.01, ***P < 0.001, compared with group S; ^#^*P* < 0.05, ^##^*P* < 0.01, ^###^*P* < 0.001, compared with the L-EEI.

Similar to the fecal microbiota, fecal metabolites differ in their sensitivity to different EEI concentrations. Figure 5 shows the effect of different EEI concentrations on fecal metabolites. A total of 73 metabolites (Supplementary Table 5) showed highly significant (*P* < 0.01, Kruskal–Wallis test) differences among the three groups. Among the top 20 metabolites in the relative abundance of identified metabolites, two metabolites [Cis-9, 10-epoxystearic acid (HMDB0247617) and (+/–)-enterolactone (HMDB0006101)] show significant differences among the three groups (*P* < 0.05). To be specific, compared with Group S, L-EEI has no significant effect on the relative abundance of Cis-9, 10-epoxystearic acid, while H-EEI could induce a significant increase compared with L-EEI (*P* < 0.01); compared with Group S, both L-EEI and H-EEI could decrease the relative abundance of (+/–)-enterolactone significantly (*P* < 0.05), while there was no significant difference between L-EEI and H-EEI.

### Many differentially expressed microorganisms are negatively correlated with metabolites

**Figure 6:**
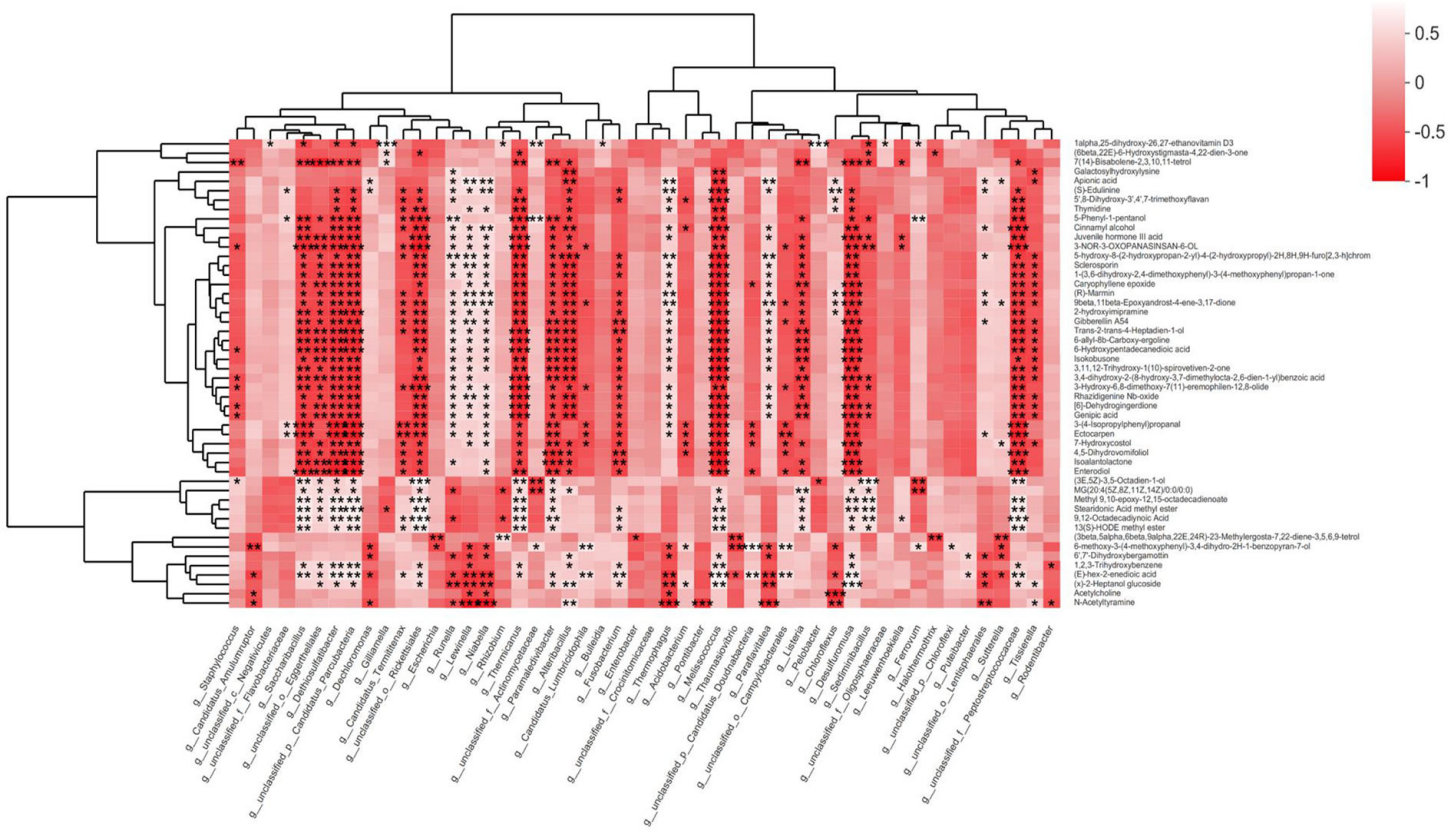
Many bacteria are negatively correlated with metabolites. The bacteria are selected using the Kruskal–Wallis test on a genus level from fecal metagenomics data, and *P* < 0.01 of them are used. The metabolites are selected using the Kruskal–Wallis test from fecal LC-MS data, and *P* < 0.01 is used. The gradient colors represent the magnitude of the correlation coefficient; red indicates a negative correlation, and a white indicates positive correlation. *P < 0.05, **P < 0.01, ***P < 0.001.

Fecal microbiota and fecal metabolite alterations are significantly related to EEI concentration. At the genus level, we linked microbiota to metabolites that were significantly different between the three groups (Figure 6). For example, g*_Sediminibacillus* had a highly significant positive correlation with (3E, 5Z)-3,5-octadien-1-ol and a substantial negative correlation with 3-3-nor-3-oxopanasinsan-6-ol. In general, most bacteria are negatively correlated with metabolites.

### Many differential metabolites are negatively correlated with metabolic pathways

**Figure 7:**
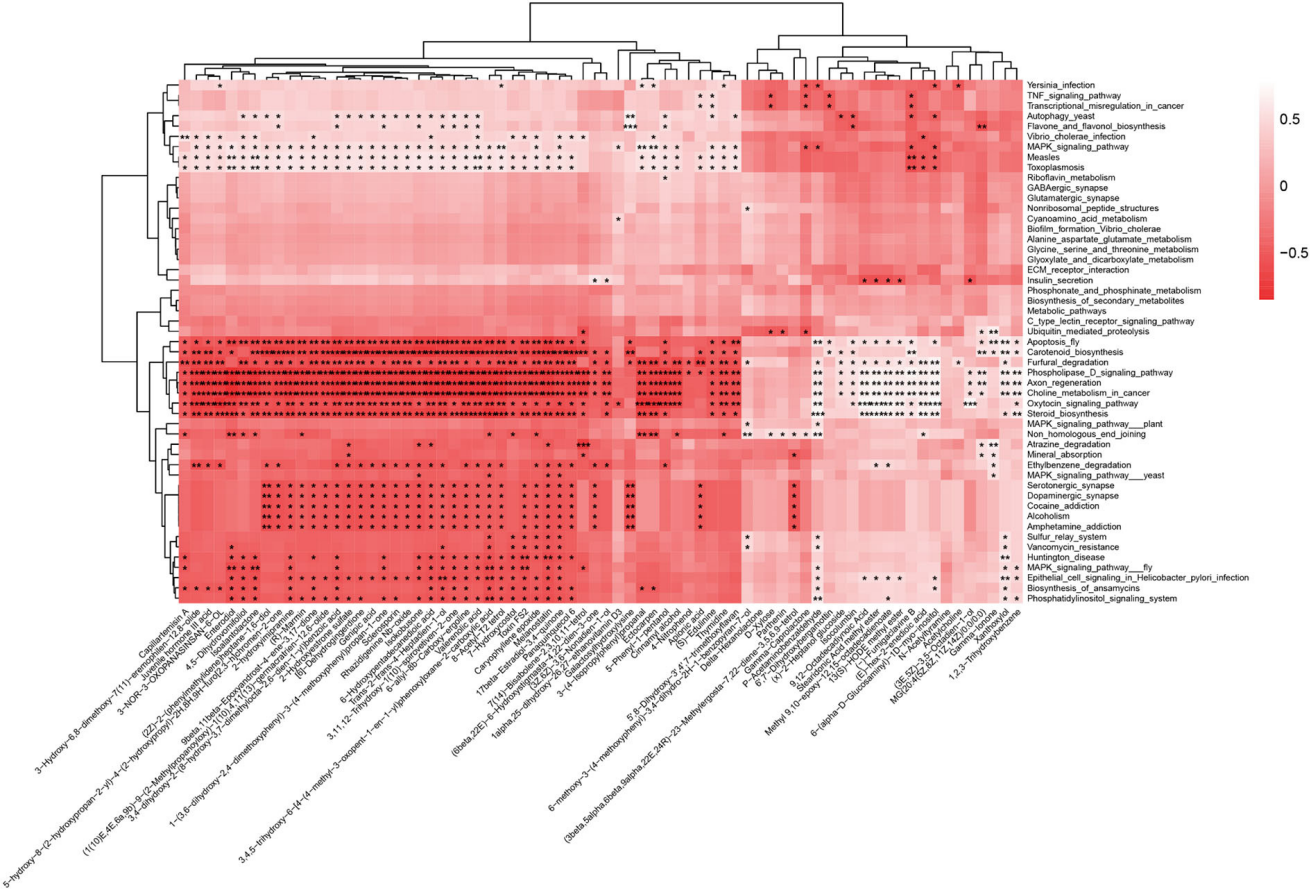
Many differential metabolites are negatively correlated with metabolic pathways. The horizontal axis represents 73 differential metabolites among three groups (*P* < 0.01) from fecal LC-MS data, and the vertical axis represents differential metabolic pathways (*P* < 0.01) among the three groups from fecal metagenomics data. The darker the color of the block, the greater the negative correlation. *P < 0.05, **P < 0.01, ***P < 0.001.

To conduct more in-depth correlation studies, we performed a metabolic pathway study on our metagenomic data and linked it to the chemicals identified in the stool samples (Figure 7). The results indicate that most differential metabolites (49/73) are negatively related to the following eight signaling pathways significantly: apoptosis (ko04214), carotenoid biosynthesis (ko00906), furfural degradation (ko00365), steroid biosynthesis (ko00100), phospholipase D signaling pathway (ko04072), axon regeneration (ko04361), choline metabolism in cancer (ko05231), and oxytocin signaling pathway (ko04921). Except for the eight signaling pathways, there are another five signaling pathways worth being concerned about: autophagy yeast, flavone and flavonol biosynthesis, MAPK signaling pathways, measles, and toxoplasmosis.

## Discussion

EEI, a Chinese anti-tumor drug derived from the traditional Chinese medicinal plant *Curcuma wenyujin*, is primarily composed of β-elemene. The main component, β-elemene, has been shown in isotope labeling studies to penetrate the blood–brain barrier (Wu et al., 2009). Its content is comparable to other tissues, with a low incidence of myelosuppression during the medication process (Chen et al., 2022). EEI has been used clinically to treat cancerous pleural effusion by pleural and cancerous ascites by intraperitoneal perfusion (Luo et al., 2019; Qureshi et al., 2019; Zhai et al., 2019), but few studies have examined its impact on fecal microbiota.

EEI alters the fecal microbiota's β-diversity but not the α-diversity. This indicates that EEI could alter fecal microbiota structure. Following EEI administration, some of the previously dominant microbiota are no longer dominant, and new dominant microbiota emerge. For example, the *Candidatus Amulumruptor* of *Muribaculaceae* was the most prominent bacterium in the H-EEI (LEfSe). The function of *Candidatus Amulumruptor* has not yet been reported. *Muribaculaceae* members use mucin monosaccharide (Pereira et al., 2020) and are abundant in the mice that had been fed an HFD (Liu et al., 2021).

*Ruminococcus bromii* and *Dorea sp. 5-2* are particularly interesting because L-EEI significantly increased their abundance while H-EEI decreased it. In our previous study, *Ruminococcus* was the dominant bacterium in the EEI group using 16S rRNA technology (https://www.ncbi.nlm.nih.gov/sra/PRJNA821627), but the impact of *Ruminococcus* on human health is complex. Its benefits include the following: *Ruminococcus albus* has an inverse relationship with ulcerative colitis (Li et al., 2020) and protects infants from allergies (Wang et al., 2021). The harmful aspects include the association of *Ruminococcus gnavus* with Crohn's disease, which has been identified as causing Crohn's symptoms (Henke et al., 2019). In addition, *Ruminococcus* has been linked to irritable bowel syndrome (Baumgartner et al., 2021). In this study, we used metagenomic technology to confirm that L-EEI (20 mg/kg·d) increased the abundance of *Ruminococcus bromii*. According to current research, *Ruminococcus bromii* is a type of bacteria beneficial to humans; this bacterium can produce short-chain fatty acids and thus alleviate type 2 diabetic symptoms (Lordan et al., 2020; Yao et al., 2020). There are currently no reports on the function of *Dorea sp. 5-2*, and most studies focus on *Dorea*. The impact of *Dorea* on human health is unclear, but studies have shown that *Dorea* is more abundant in patients with Parkinson's (Petrov et al., 2017) and obesity (Jiao et al., 2018). It is unknown why the abundance of *Ruminococcus bromii* and *Dorea sp. 5-2* significantly increased at L-EEI but decreased at H-EEI. This is also one of the future topics we intend to investigate.

Fecal metabolomics is the consequence of both host and microbiota interaction. Figure 5 displays the effect of different concentrations of EEI on metabolites. A total of 73 metabolites had extremely significant statistical differences among the three groups (*P* < 0.01). In the abundance of the top 20 differential metabolites, two kinds of metabolites [Cis-9,10-Epoxystearic acid and (+/–)-Enterolactone] displayed significant statistical differences among the three groups (*P* < 0.05). Compared to L-EEI, H-EEI had a higher Cis-9,10-epoxystearic acid content. The effects of Cis-9,10-Epoxystearic acid on humans are unclear. Cis-9,10-Epoxystearic acid dose- and time-dependently increased the number and size of cellular lipid droplets in the human hepatocarcinoma cell line HepG2, decreasing cell viability and causing cell death (Liu et al., 2018; Liu Y. et al., 2020). Compared to group S, both L-EEI and H-EEI could decrease the abundance of (+/–)-enterolactone significantly (*P* < 0.05). Enterolactone's health implications are debated. Enterolactone, a bioactive phenolic metabolite from dietary lignans, may help protect against different stages of breast, prostate, colon, gastric, and lung cancer (Mali et al., 2019; Senizza et al., 2020). Unfortunately, a case-control study found no association between serum EL levels and breast cancer risk (Kilkkinen et al., 2004). A New York prospective study found comparable outcomes (Zeleniuch-Jacquotte et al., 2004). β-elemene partially corrected HFD-induced alterations in mouse gut bacteria composition and metabolites (Zhou et al., 2021). Because EEI is used clinically for intraperitoneal infusion therapy of cancerous ascites, we examined its effects on rat fecal microbiota and metabolites (Jiang et al., 2012; Zhu et al., 2019).

In addition to fecal microbiota, we used 16S rRNA technology to investigate the effects of different EEI concentrations on colon contents. EEI concentrations, like fecal microbiota, did not affectα-diversity of colonic microbiota (Supplementary Table 6). Genus-level community composition is shown in Supplementary Figure 4B. The NMDS analysis of different groups revealed distinct genus-level clusters. Similar to fecal microbiota, L-EEI and group S differed less than H-EEI, and group S. Group S and H-EEI stood out visually (Supplementary Figure 4D). Unlike the fecal microbiota, the most prominent biomarkers in the colon contents of low and high elemene groups were g*_Marvinbryantia* and g*_norank_*f*_Erysipelotrichaceae*, respectively (Supplementary Figure 5, LEfSe).

IP injection of EEI alters the microbiota and metabolites in rat feces. Since the gut microbiota is linked to several cancers and EEI inhibits the growth of various cancers, we hope to research the effect of EEI on the feces of patients with cancer.

## Data availability statement

The datasets presented in this study can be found in online repositories. The names of the repository/repositories and accession number(s) can be found below: https://www.ncbi.nlm.nih.gov/, PRJNA821627 and https://www.ncbi.nlm.nih.gov/sra/PRJNA866381.

## Ethics statement

The animal study was reviewed and approved by Animal Care and Use Committee of Air Force Medical University. The approval number is: IACUC-20220522.

## Author contributions

JZ, LM, and TL designed the study and approved the final manuscript version. LG and HW performed the experiments and wrote the manuscript. YZ, YJ, and YW analyzed the data and participated in the metagenomic exploration. All authors have read and approved the final manuscript.

## Funding

This study was supported by the Science and Technology Innovation Development Fund of the Second Affiliated Hospital of the Air Force Military Medical University (2019LCYJ012 to JZ), Key Clinical Research Projects of the Second Affiliated Hospital of the Air Force Military Medical University (2021LCYJ016 to JZ), the Social Talent Funding Program Project of the Second Affiliated Hospital of the Air Force Military Medical University (2021SHRC068 to HW), and Key Scientific Research Project of TCM Inheritance, Innovation of Qin Medicine Development in 2021 (2021-01-ZZ-006).

## Conflict of interest

The authors declare that the research was conducted in the absence of any commercial or financial relationships that could be construed as a potential conflict of interest.

## Publisher's note

All claims expressed in this article are solely those of the authors and do not necessarily represent those of their affiliated organizations, or those of the publisher, the editors and the reviewers. Any product that may be evaluated in this article, or claim that may be made by its manufacturer, is not guaranteed or endorsed by the publisher.
