# Supplementary material for: The effects of elemene emulsion injection on rat fecal microbiota and metabolites: Evidence from metagenomic exploration and liquid chromatography-mass spectrometry
Source: Front Microbiol. 2022 Nov 24;13:913461. doi: 10.3389/fmicb.2022.913461 (PMC9730252; doi:10.3389/fmicb.2022.913461)
Supplement: Supplementary file 1 [file Table_1.pdf]

Supplementary Table 1. Raw sequence statistics (columns 2-5), statistics after quality control (columns 6-9) and statistics table after decontaminating the host genome (10-13). The data used in the analysis were derived from metagenomics sequencing data of fecal mirobiota.

Samples	Insert Size (bp)	Read length (bp)	Raw reads	Raw base (bp)	Clean reads	Clean base(bp)	Percent in raw reads (%)	Percent in raw bases (%)	Optimized reads	Optimized bases (bp)	Percent in raw reads (%)	Percent in raw bases (%)
S1	500	150	54009338	8155410038	53230918	8029997405	98.56	98.46	40015818	6039337839	74.09	74.05
S2	500	150	43749236	6606134636	43119628	6505241119	98.56	98.47	31734666	4789687414	72.54	72.50
S3	500	150	44608046	6735814946	43606922	6573461791	97.76	97.59	28439388	4291062770	63.75	63.71
S4	500	150	46350322	6998898622	45547644	6869498629	98.27	98.15	34565408	5215887588	74.57	74.52
S5	500	150	52411956	7914205356	51694852	7796059156	98.63	98.51	37652906	5681922453	71.84	71.79
S6	500	150	42204076	6372815476	41530182	6264451409	98.40	98.30	31793846	4798019088	75.33	75.29
L1	500	150	49092700	7412997700	48500942	7315795784	98.79	98.69	35813858	5405176182	72.95	72.91
L2	500	150	46535118	7026802818	45362046	6831370013	97.48	97.22	9147468	1380501849	19.66	19.65
L3	500	150	54250220	8191783220	53694730	8100524702	98.98	98.89	43094416	6503968075	79.44	79.40
L4	500	150	49625132	7493394932	48999542	7391861906	98.74	98.65	37643296	5681217474	75.86	75.82
L5	500	150	43869952	6624362752	43036988	6491359419	98.10	97.99	32461614	4898645881	74.00	73.95
L6	500	150	45399504	6855325104	44681118	6738093022	98.42	98.29	31157552	4701868938	68.63	68.59
H1	500	150	52065724	7861924324	51168106	7717747923	98.28	98.17	38899988	5870156733	74.71	74.67
H2	500	150	49189918	7427677618	48474240	7306980116	98.55	98.38	27580616	4161943153	56.07	56.03
H3	500	150	46495438	7020811138	45902304	6921377999	98.72	98.58	32745180	4941041192	70.43	70.38
H4	500	150	40927274	6180018374	40181200	6051401406	98.18	97.92	13737962	2073331999	33.57	33.55
H5	500	150	46455830	7014830330	45849452	6915685045	98.69	98.59	33464448	5050455743	72.03	72.00
H6	500	150	46950154	7089473254	46290132	6983121007	98.59	98.50	36641458	5529917715	78.04	78.00
