# Supplementary material for: The effects of elemene emulsion injection on rat fecal microbiota and metabolites: Evidence from metagenomic exploration and liquid chromatography-mass spectrometry
Source: Front Microbiol. 2022 Nov 24;13:913461. doi: 10.3389/fmicb.2022.913461 (PMC9730252; doi:10.3389/fmicb.2022.913461)
Supplement: Supplementary file 2 [file Table_2.pdf]

Supplementary Table 2. Statistics table of assembly result from metagenomics sequencing data of fecal mirobiota

Sample	ORFs	Total Length (bp)	Average Length (bp)	Max (bp)	Min (bp)
S1	492897	268420491	544.58	27231	102
S2	374827	206584524	551.15	67134	102
S3	361281	196246809	543.2	16782	102
S4	505375	268273869	530.84	40527	102
S5	400317	214058943	534.72	16782	102
S6	399368	217915164	545.65	40527	102
L1	430720	235341867	546.39	31596	102
L2	161268	84677826	525.08	15507	102
L3	466751	252346251	540.64	32040	102
L4	447781	249222249	556.57	126831	102
L5	497830	274441026	551.27	35151	102
L6	437096	241011327	551.39	116004	102
H1	616945	342186462	554.65	23856	102
H2	356622	200890920	563.32	28302	102
H3	380791	220442313	578.91	23790	102
H4	192121	110026608	572.69	14625	102
H5	343198	197103744	574.31	40089	102
H6	406310	235690521	580.08	93063	102
