# Supplementary material for: The effects of elemene emulsion injection on rat fecal microbiota and metabolites: Evidence from metagenomic exploration and liquid chromatography-mass spectrometry
Source: Front Microbiol. 2022 Nov 24;13:913461. doi: 10.3389/fmicb.2022.913461 (PMC9730252; doi:10.3389/fmicb.2022.913461)
Supplement: Supplementary file 3 [file Table_3.pdf]

Supplementary Table 3. Gene prediction result statistics table from metagenomics sequencing data of fecal mirobiota

Sample	Contigs	Contigs bases (bp)	N50 (bp)	N90 (bp)	Max (bp)	Min (bp)
S1	303586	303457773	1429	399	268661	300
S2	229904	233231009	1510	400	572895	300
S3	225851	221622992	1357	395	260241	300
S4	328557	302433937	1142	391	276147	300
S5	257365	241953021	1224	388	489220	300
S6	253458	245124410	1243	395	215091	300
L1	269712	266020914	1329	398	481695	300
L2	107080	95400800	1021	391	266016	300
L3	293685	285631504	1301	394	443642	300
L4	276305	280958600	1402	410	302226	300
L5	307098	309889944	1402	401	361852	300
L6	272410	271749129	1403	402	291193	300
H1	383472	385936324	1401	406	391251	300
H2	222729	225236229	1476	398	547802	300
H3	230335	250409186	1810	409	250296	300
H4	119627	123512473	1521	405	219108	300
H5	203134	221808080	1783	411	643045	300
H6	239096	265391040	1798	417	281739	300
