# Supplementary material for: The effects of elemene emulsion injection on rat fecal microbiota and metabolites: Evidence from metagenomic exploration and liquid chromatography-mass spectrometry
Source: Front Microbiol. 2022 Nov 24;13:913461. doi: 10.3389/fmicb.2022.913461 (PMC9730252; doi:10.3389/fmicb.2022.913461)
Supplement: Supplementary file 4 [file Table_4.pdf]

Supplementary Table 4. Alpha diversity of fecal microbiota from metagenomics sequencing data of fecal mirobiota

sample	ace	shannon	simpson	chao	shannoneven
S1	9791	5.671954	0.013356	9791	0.61724
S2	8944	5.546967	0.013431	8944	0.609641
S3	9189	5.427924	0.016905	9189	0.594791
S4	9457	5.377663	0.022426	9457	0.587433
S5	9374	5.241757	0.021648	9374	0.573139
S6	9255	5.34869	0.020649	9255	0.58565
L1	8899	4.991051	0.04286	8899	0.548847
L2	7040	5.350166	0.019211	7040	0.6039
L3	9067	4.891966	0.048785	9067	0.536847
L4	9492	5.606978	0.012747	9492	0.612235
L5	9523	5.725784	0.012654	9523	0.624986
L6	9392	5.689886	0.012062	9392	0.622008
H1	9333	5.654383	0.016859	9333	0.618553
H2	8790	5.38327	0.016127	8790	0.592782
H3	8895	5.290788	0.019579	8895	0.581837
H4	7144	5.096372	0.022325	7144	0.574302
H6	9114	5.483854	0.014469	9114	0.60146
H5	8705	5.228986	0.024017	8705	0.576409
