# Supplementary material for: The effects of elemene emulsion injection on rat fecal microbiota and metabolites: Evidence from metagenomic exploration and liquid chromatography-mass spectrometry
Source: Front Microbiol. 2022 Nov 24;13:913461. doi: 10.3389/fmicb.2022.913461 (PMC9730252; doi:10.3389/fmicb.2022.913461)
Supplement: Supplementary file 5 [file Table_5.pdf]

Supplementary Table 5. A total of 73 metabolites showed highly significant (*P* < 0.01, Kruskal-Wallis test) differences among the three groups. The data used in the analysis were derived from LC-MS data of fecal metabolites.

Metabolite	Sham_mean	Sham_SD	Low_mean	Low_SD	High_mean	High_SD	*P*
7(14)-Bisabolene-2,3,10,11-tetrol	4.782	0.1326	5.16	0.1861	5.01	0.1866	0.009683
Galactosylhydroxylysine	4.801	0.125	4.988	0.1234	5.062	0.03121	0.009683
3-(4-Isopropylphenyl)propanal	4.793	0.09836	4.907	0.04452	4.936	0.02649	0.009514
P-Acetaminobenzaldehyde	4.683	0.1921	4.512	0.187	4.304	0.1216	0.009514
N-Acetyltyramine	5.756	0.06809	5.684	0.112	5.532	0.1073	0.008715
6-methoxy-3-(4-methoxyphenyl)-3,4-dihydro-2H-1-benzopyran-7-ol	5.261	0.02952	5.279	0.07223	5.183	0.0292	0.008317
(3beta,5alpha,6beta,9alpha,22E,24R)-23-Methylergosta-7,22-diene-3,5,6,9-tetrol	6.041	0.06488	6.12	0.01089	6.03	0.07951	0.008317
Ectocarpen	4.505	0.1015	4.622	0.03882	4.663	0.02708	0.008317
Parthenin	3.928	0.1083	3.977	0.09255	3.762	0.05948	0.007486
1alpha,25-dihydroxy-26,27-ethanovitamin D3	5.831	0.05374	5.912	0.03793	5.822	0.0392	0.007486
13(S)-HODE methyl ester	5.705	0.143	5.355	0.1301	5.498	0.1387	0.007186
(3Z,6Z)-3,6-Nonadien-1-ol	4.11	0.09327	4.325	0.09931	4.294	0.06666	0.007061
Acetylcholine	5.936	0.2579	5.741	0.07191	5.396	0.2619	0.006979
(3E,5Z)-3,5-Octadien-1-ol	5.043	0.05061	4.965	0.02811	5.026	0.02069	0.006281
Gamma-Caprolactone	3.693	0.1325	3.737	0.08516	3.559	0.04163	0.006065
4-Nitrophenol	3.921	0.1059	4.103	0.2198	4.428	0.306	0.005925
Gamma-Ionone	3.614	0.1661	3.133	0.2684	3.478	0.1771	0.005523
Methyl 9,10-epoxy-12,15-octadecadienoate	6.386	0.1583	5.981	0.1283	6.162	0.1098	0.005179
6',7'-Dihydroxybergamottin	4.814	0.1174	4.706	0.1105	4.589	0.05919	0.005089
(x)-2-Heptanol glucoside	4.729	0.07569	4.609	0.04515	4.556	0.06667	0.004971
MG(20:4(5Z,8Z,11Z,14Z)/0:0/0:0)	6.245	0.1208	5.928	0.1382	6.079	0.06992	0.004828
Enterodiol	4.295	0.2903	4.758	0.09659	4.909	0.1702	0.004828
Cinnamyl alcohol	4.491	0.07668	4.62	0.06181	4.626	0.05077	0.00458
Delta-Hexanolactone	3.73	0.05197	3.788	0.1033	3.578	0.04896	0.00432
Isocolumbin	4.18	0.2009	3.818	0.2108	3.604	0.117	0.004171
9,12-Octadecadiynoic Acid	6.096	0.145	5.748	0.09214	5.884	0.1143	0.003625
1,2,3-Trihydroxybenzene	5.663	0.01765	5.622	0.01916	5.611	0.02222	0.003625
Stearidonic Acid methyl ester	5.403	0.1107	5.108	0.09069	5.218	0.08039	0.003562
6-(alpha-D-Glucosaminyl)-1D-myo-inositol	4.35	0.1716	3.996	0.137	3.897	0.1253	0.00334
Juvenile hormone III acid	5.613	0.08354	6.099	0.1053	6.118	0.0987	0.003225
3-NOR-3-OXOPANASINSAN-6-OL	4.735	0.2343	5.481	0.141	5.456	0.1209	0.003225
5-Phenyl-1-pentanol	4.492	0.07571	4.735	0.1099	4.685	0.0215	0.003225
3,4-dihydroxy-2-(8-hydroxy-3,7-dimethylocta-2,6-dien-1-yl)benzoic acid	3.814	0.07062	5.236	0.1405	5.303	0.1516	0.002754
4,5-Dihydrovomifoliol	4.822	0.06537	4.916	0.01955	4.957	0.05244	0.002706
Genipic acid	3.553	0.097	5.197	0.05424	5.338	0.1645	0.002552
(6beta,22E)-6-Hydroxystigmasta-4,22-dien-3-one	5.058	0.05763	5.239	0.07232	5.143	0.06365	0.002464
D-Xylose	3.257	0.03657	3.344	0.1611	2.99	0.06683	0.002436
Xanthoxylol	4.362	0.1706	3.953	0.161	3.795	0.161	0.002436
Thymidine	4.71	0.04706	4.896	0.06032	4.985	0.09887	0.002338
Apionic acid	4.77	0.02221	4.807	0.03038	4.856	0.01149	0.001861
5',8-Dihydroxy-3',4',7-trimethoxyflavan	5.331	0.04081	5.447	0.05946	5.525	0.06081	0.001685
Isokobusone	5.102	0.09287	5.67	0.09589	5.805	0.1102	0.001675
6-Hydroxypentadecanedioic acid	4.284	0.106	5.238	0.08826	5.391	0.139	0.001464
Trans-2-trans-4-Heptadien-1-ol	4.467	0.08034	5.114	0.1386	5.264	0.07764	0.001464
8-Acetyl-T2 tetrol	2.801	0.264	3.978	0.09765	4.166	0.1506	0.001464
3-Hydroxy-6,8-dimethoxy-7(11)-eremophilen-12,8-olide	4.691	0.1989	5.625	0.08126	5.737	0.07711	0.001464
2-Hydroxyestrone sulfate	1.985	0.1963	4.337	0.1318	4.593	0.1733	0.001265
Isoalantolactone	4.781	0.08215	4.962	0.02834	5.017	0.05662	0.001265
Caryophyllene epoxide	4.21	0.03878	4.878	0.2013	5.1	0.1458	0.001265
(S)-Edulinine	4.658	0.04346	4.816	0.07783	4.969	0.08951	0.001139
3,4,5-trihydroxy-6-[4-(4-methyl-3-oxopent-1-en-1-yl)phenoxy]oxane-2-carboxylic acid	2.952	0.3939	4.202	0.1746	4.507	0.1548	0.00108
Melanostatin	1.723	0.1834	3.308	0.2377	3.789	0.279	0.00108
7-Hydroxycostol	4.302	0.0917	4.601	0.06728	4.801	0.143	0.00108
Panaquinquecol 6	3.227	0.05917	4.257	0.1939	4.586	0.2441	0.00108
[6]-Dehydrogingerdione	3.726	0.05872	5.493	0.06746	5.694	0.1346	0.0009119
3,11,12-Trihydroxy-1(10)-spirovetiven-2-one	4.788	0.08821	5.156	0.06963	5.27	0.0605	0.0009119
(E)-hex-2-enedioic acid	6.218	0.02	6.173	0.01846	6.132	0.01524	0.0007742
(R)-Marmin	4.44	0.03561	4.915	0.09945	5.148	0.1131	0.0007607

2-hydroxyimipramine	4.513	0.2058	5.211	0.02867	5.48	0.1308	0.0006272
Valerenolic acid	2.748	0.1423	3.83	0.07457	4.102	0.1679	0.0006272
17beta-Estradiol-3,4-quinone	3.375	0.09482	4.179	0.1787	4.524	0.1899	0.0006272
6-allyl-8b-Carboxy-ergoline	3.899	0.217	5.685	0.1182	5.977	0.09335	0.0005111
Rhazidigenine Nb-oxide	3.803	0.04609	5.23	0.08387	5.504	0.1563	0.0005111
5-hydroxy-8-(2-hydroxypropan-2-yl)-4-(2-hydroxypropyl)-2H,8H,9H-furo[2,3-h]chromen-2-one	3.887	0.07341	4.71	0.09243	5.005	0.09526	0.0005111
Toxin FS2	3.375	0.1711	4.248	0.08834	4.818	0.3142	0.0005111
(-)-Fumigaclavine B	3.961	0.1418	3.402	0.08545	3.126	0.07371	0.0005111
Sclerosporin	4.262	0.0138	5.322	0.06054	5.603	0.07935	0.0005111
Gibberellin A54	3.938	0.1368	5.035	0.1018	5.414	0.09715	0.0005111
9beta,11beta-Epoxyandrost-4-ene-3,17-dione	4.271	0.06072	4.878	0.04176	5.204	0.09475	0.0005111
1-(3,6-dihydroxy-2,4-dimethoxyphenyl)-3-(4-methoxyphenyl)propan-1-one	3.746	0.08273	4.954	0.1007	5.266	0.09549	0.0005111
(2Z)-2-(phenylmethylidene)heptane-1,6-diol	4.073	0.08434	4.607	0.05842	4.813	0.07351	0.0005111
(1(10)E,4E,6a,9b)-9-(2-Methylpropanoyloxy)-1(10),4,11(13)-germacratrien-12,6-olide	3.713	0.03434	4.43	0.04455	4.719	0.09159	0.0005111
Capillartemisin A	3.712	0.06937	3.945	0.07182	4.273	0.2229	0.0005111
