# Supplementary material for: The effects of elemene emulsion injection on rat fecal microbiota and metabolites: Evidence from metagenomic exploration and liquid chromatography-mass spectrometry
Source: Front Microbiol. 2022 Nov 24;13:913461. doi: 10.3389/fmicb.2022.913461 (PMC9730252; doi:10.3389/fmicb.2022.913461)
Supplement: Supplementary file 6 [file Table_6.pdf]

Supplementary Table 6. The Alpha diversity of colonic contents performed on 16S rRNA data of colonic contents.

	High-Mean	High-Sd	Low-Mean	Low-Sd	*P*
ace	504.33	23.787	500.8	48.259	0.9362
chao	519.45	12.339	504.78	53.749	0.9362
coverage	0.99751	0.0004159	0.99793	0.00058597	0.1735
shannon	4.0028	0.44015	3.9261	0.47107	0.9362
simpson	0.054493	0.033292	0.061505	0.034501	0.8102
sobs	442.83	26.095	441.83	41.778	0.8099
