# Supplementary material for: The effects of elemene emulsion injection on rat fecal microbiota and metabolites: Evidence from metagenomic exploration and liquid chromatography-mass spectrometry
Source: Front Microbiol. 2022 Nov 24;13:913461. doi: 10.3389/fmicb.2022.913461 (PMC9730252; doi:10.3389/fmicb.2022.913461)

Supplementary Figure 1. The relative abundance of the species and families in each group. The relative abundance of the species (A) and family (B) in each group was performed on fecal metagenomics data.

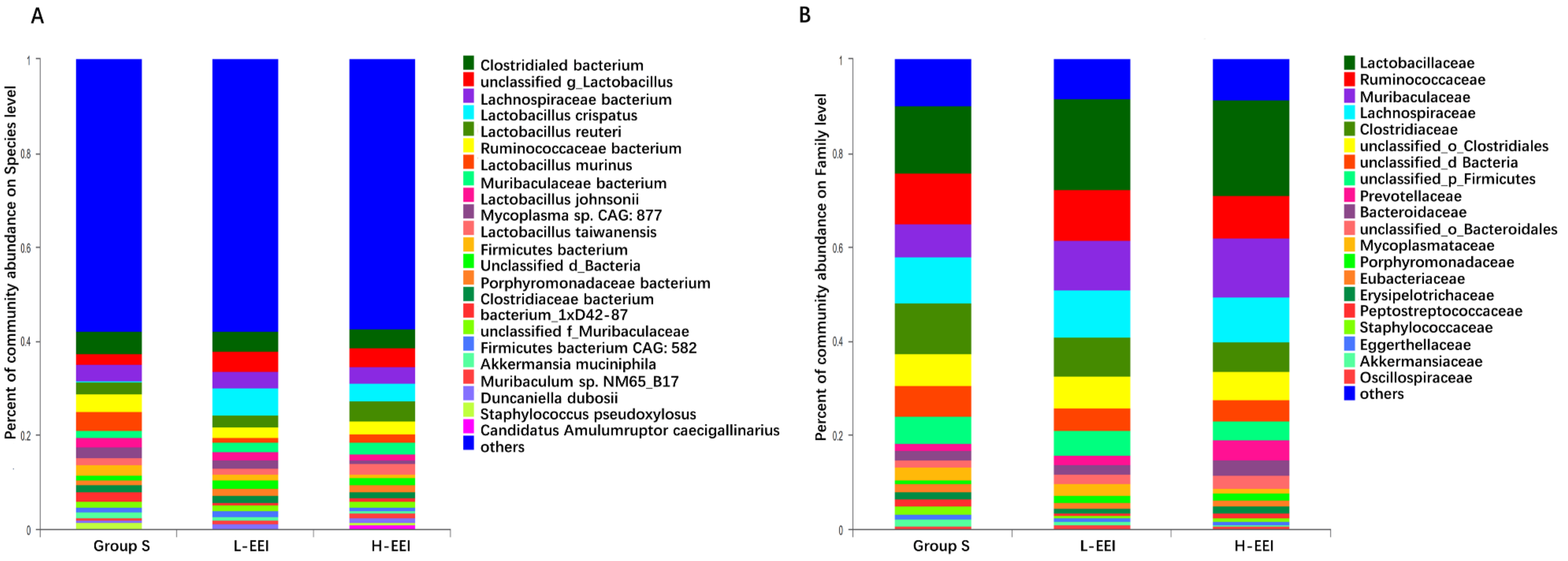

Supplement: Supplementary file 7 [file Image_1.pdf]
