# Supplementary material for: The effects of elemene emulsion injection on rat fecal microbiota and metabolites: Evidence from metagenomic exploration and liquid chromatography-mass spectrometry
Source: Front Microbiol. 2022 Nov 24;13:913461. doi: 10.3389/fmicb.2022.913461 (PMC9730252; doi:10.3389/fmicb.2022.913461)

Supplementary Figure 2. Statistical validity analysis of fecal LC-MS data. A: PLS-DA principal component number selection. R2Y and Q2 are used to evaluate the explanatory and prediction abilities of the PLS-DA model, respectively. The higher the cumulative value of R2Y and Q2, the more stable and reliable the model. B: PLS-DA model validation. The abscissa represents the permutation retention of the permutation test (the proportion that is consistent with the order of the Y variable of the original model; the point with the permutation retention of 1 is the R2 and Q2 values of the original model); and the ordinate represents the R2 (red dots) and Q2 (blue triangles) values of the permutation test, and the two dashed lines represent the regression lines for R2 and Q2, respectively. A total of 200 random permutation tests were chosen. The intercept of the Q2 regression line and the Y axis is less than 0.05, indicating that the model is robust and reliable with no overfitting.

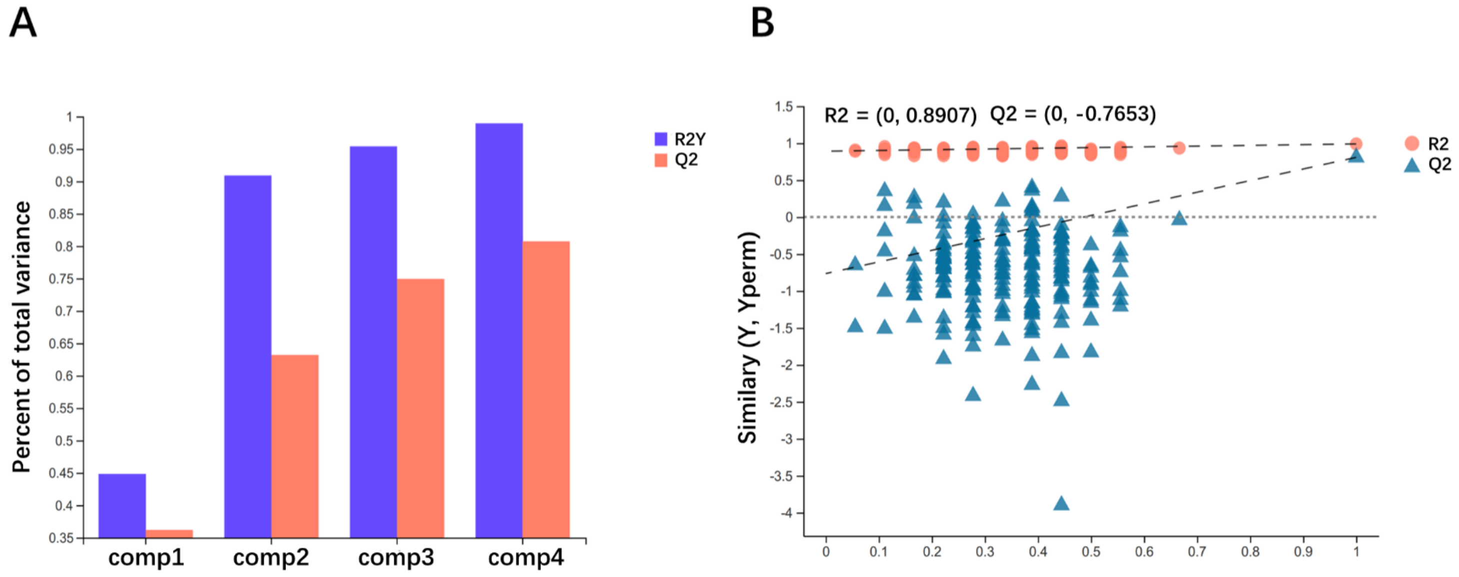

Supplement: Supplementary file 8 [file Image_2.pdf]
