# Supplementary material for: The effects of elemene emulsion injection on rat fecal microbiota and metabolites: Evidence from metagenomic exploration and liquid chromatography-mass spectrometry
Source: Front Microbiol. 2022 Nov 24;13:913461. doi: 10.3389/fmicb.2022.913461 (PMC9730252; doi:10.3389/fmicb.2022.913461)

Supplementary Figure 3. The total ion chromatograms of the quality control samples in positive (A) and negative (B) ion mode. The chromatographically separated components enter the mass spectrometer continuously, and the mass spectrometer is scanned for data collection continuously. Each scan yields a mass spectrum, and the sum of all ion intensities in each mass spectrum yields the total ion current intensity. The total ion chromatogram is drawn with time as the abscissa and total ion intensity as the ordinate. The data used in the analysis were derived from LC-MS data of fecal metabolites.

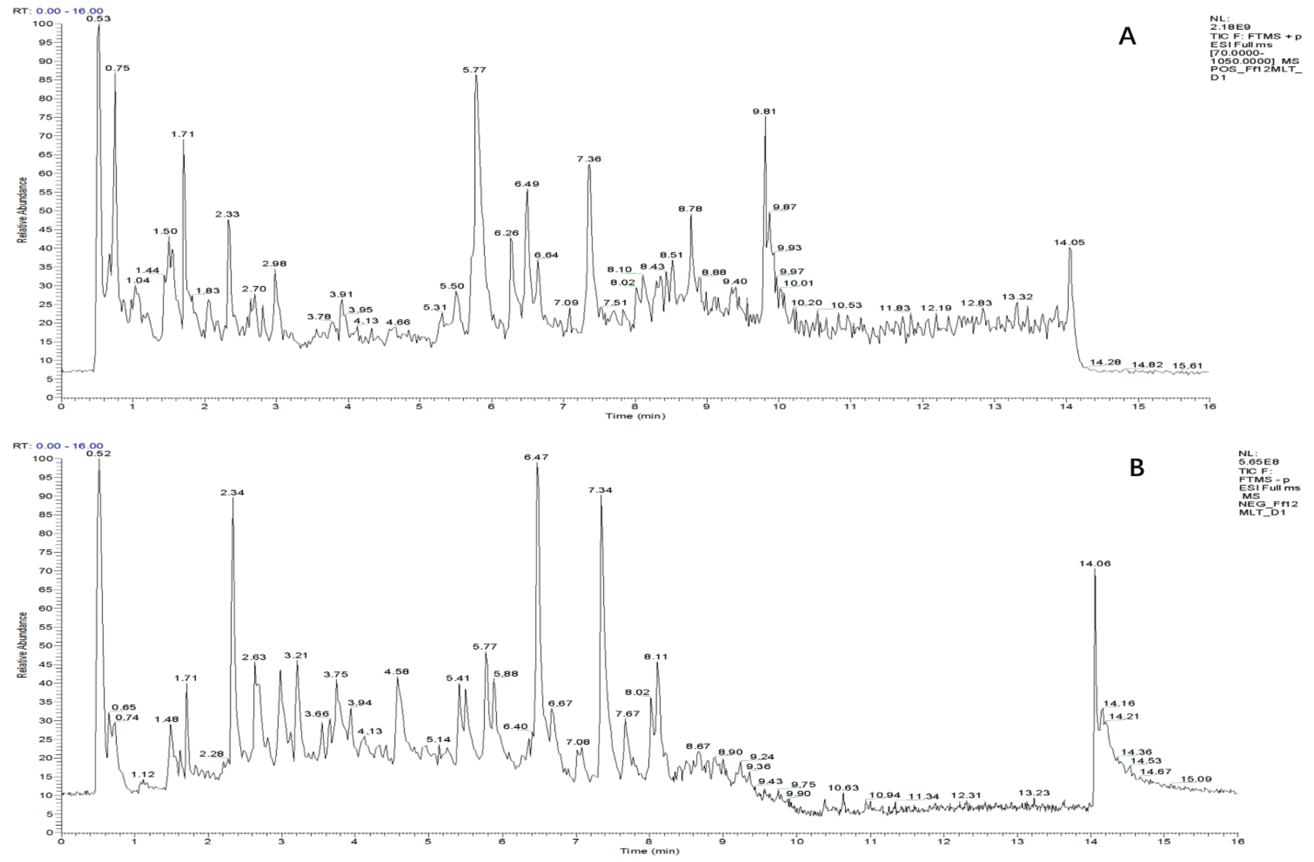

Supplement: Supplementary file 9 [file Image_3.pdf]
