# Supplementary material for: The effects of elemene emulsion injection on rat fecal microbiota and metabolites: Evidence from metagenomic exploration and liquid chromatography-mass spectrometry
Source: Front Microbiol. 2022 Nov 24;13:913461. doi: 10.3389/fmicb.2022.913461 (PMC9730252; doi:10.3389/fmicb.2022.913461)

Supplementary Figure 4. Different concentration of EEI altered the β-diversity of colonic contents. A: The Venn diagrams shows that bacteria are unique and shared by different groups at the genus level. B: The relative abundance of the genus level in each group. C: Similarity test among the three groups (PERMANOVA, R2 = 0.174; *P* = 0.035). D: NMDS analysis shows a distinct formation of clusters at the genus level (stress: 0.122). The data used in the analysis were derived from 16S rRNA of colonic contents, n = 6 in each group.

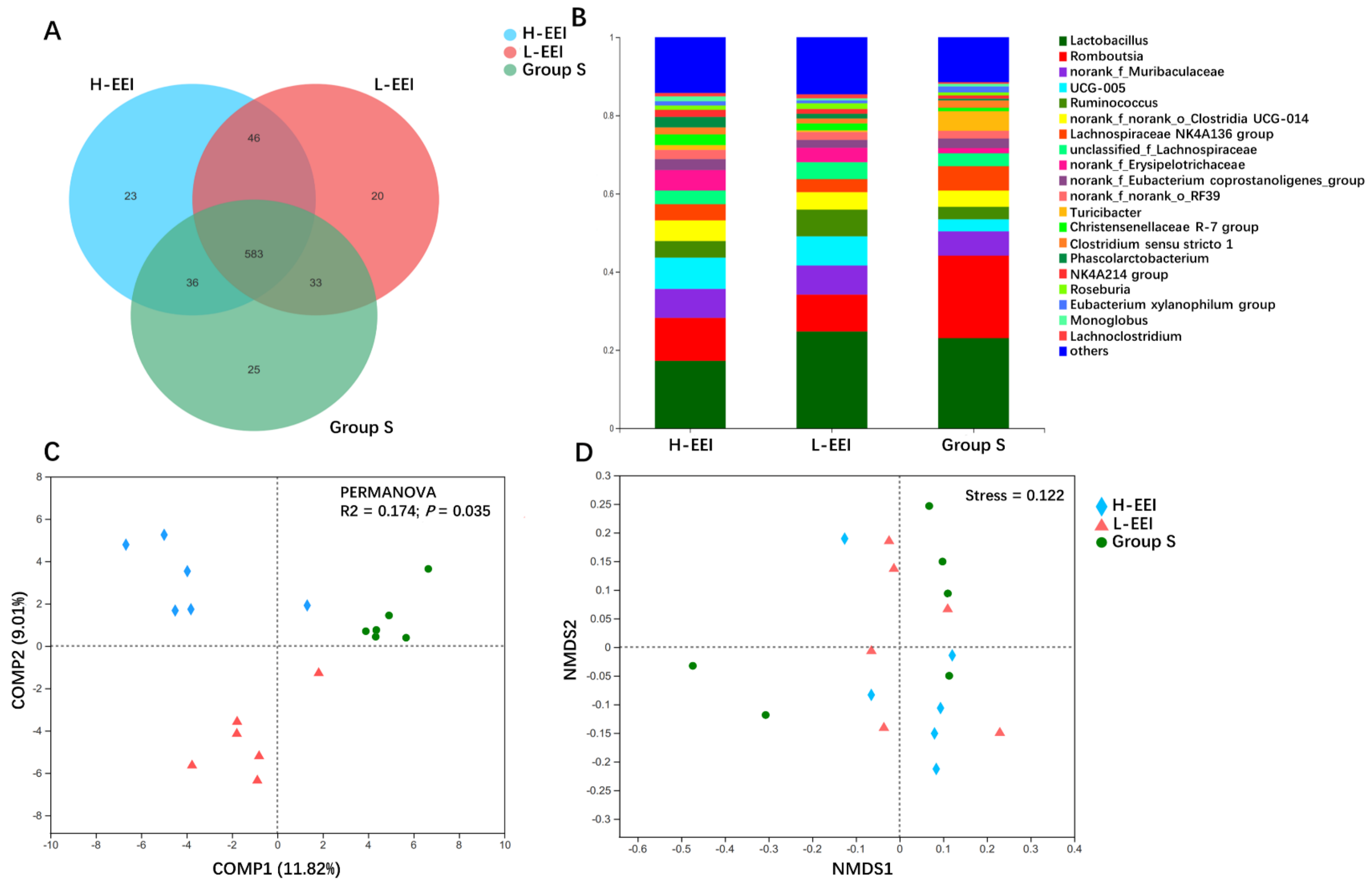

Supplement: Supplementary file 10 [file Image_4.pdf]
