# Supplementary material for: The effects of elemene emulsion injection on rat fecal microbiota and metabolites: Evidence from metagenomic exploration and liquid chromatography-mass spectrometry
Source: Front Microbiol. 2022 Nov 24;13:913461. doi: 10.3389/fmicb.2022.913461 (PMC9730252; doi:10.3389/fmicb.2022.913461)

Supplementary Figure 5. Biomarker of colonic contents in different EEI concentration. A: The hierarchy of LEfSe was performed on 16S rRNA data of colonic contents. B: The linear discriminant analysis (LDA) discriminant histogram. The larger the LDA score, the greater the influence of species abundance on the differential effect. *P* < 0.05, LDA > 3.0

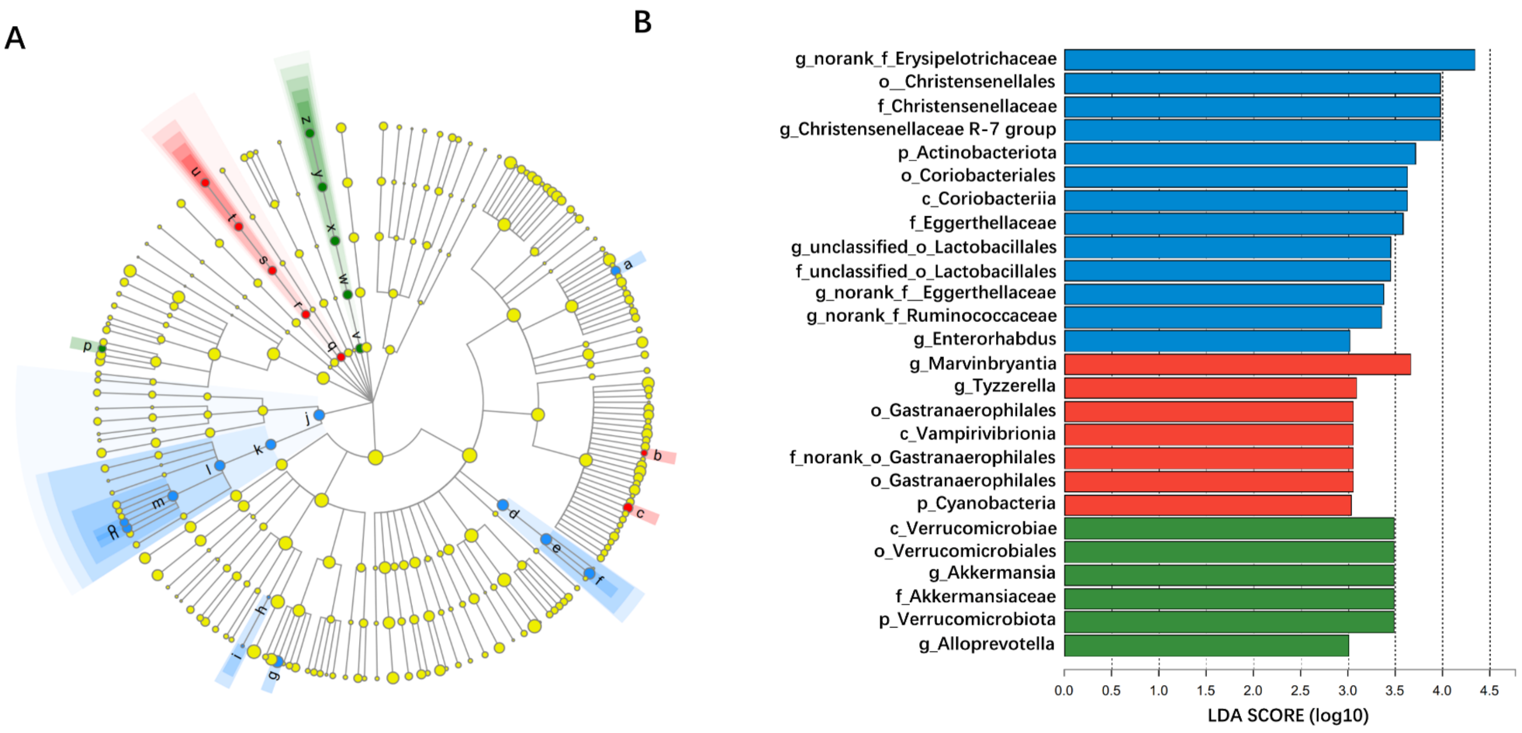

Supplement: Supplementary file 11 [file Image_5.pdf]
